# Virulence of *Hymenoscyphus albidus* and *H*. *fraxineus* on *Fraxinus excelsior* and *F*. *pennsylvanica*

**DOI:** 10.1371/journal.pone.0141592

**Published:** 2015-10-30

**Authors:** Tadeusz Kowalski, Piotr Bilański, Ottmar Holdenrieder

**Affiliations:** 1 Department of Forest Pathology, Mycology and Tree Physiology, University of Agriculture, Cracow, Poland; 2 Department of Forest Protection, Forest Entomology and Climatology, University of Agriculture, Cracow, Poland; 3 Forest Pathology and Dendrology, Institute of Integrative Biology, ETH Zurich, Zurich, Switzerland; Natural Resources Canada, CANADA

## Abstract

European ash (*Fraxinus excelsior*) is currently battling an onslaught of ash dieback, a disease emerging in the greater part of its native area, brought about by the introduction of the ascomycete *Hymenoscyphus fraxineus* (= *Hymenoscyphus pseudoalbidus*). The closely-related fungus *Hymenoscyphus albidus*, which is indigenous to Europe, is non-pathogenic when in contact with *F*. *excelsior*, but could pose a potential risk to exotic *Fraxinus* species. The North American green ash (*Fraxinus pennsylvanica*) is planted widely throughout Europe and regenerates naturally within this environment but little is known about the susceptibility of this species to ash dieback. We performed wound inoculations with both fungi (nine strains of *H*. *fraxineus* and three strains of *H*. *albidus*) on rachises and stems of *F*. *excelsior* and *F*. *pennsylvanica* under field conditions in Southern Poland. Necrosis formation was evaluated after two months on the rachises and after 12 months on the stems. After inoculation of *H*. *albidus*, only small lesions (of up to 1.3 cm in length) developed on the *F*. *excelsior* and *F*. *pennsylvanica* rachises, but with no significant distinction from the controls. *Hymenoscyphus albidus* did not cause necrotic lesions on the stems of either *Fraxinus* species. In contrast, *H*. *fraxineus* induced necroses on all inoculated rachises of both ash species with mean lengths of 8.4 cm (*F*. *excelsior*) and 1.9 cm (*F*. *pennsylvanica*). Necroses also developed on all of the inoculated *F*. *excelsior* stems (mean length 18.0 cm), whereas on *F*. *pennsylvanica* such lesions only occurred on about 5% of the stems (mean length 1.9 cm). The differences between strains were negligible. No necroses were observed on the control plants. Reisolations of *H*. *albidus* were only successful in around 8–11% of the cases, while *H*. *fraxineus* was reisolated from 50–70% of the inoculated organs showing necrotic lesions. None of the *Hymenoscyphus* species were isolated from the control plants. Our data confirm *H*. *fraxineus*’ high virulence with regards to *F*. *excelsior* and demonstrate a low virulence in relation to *F*. *pennsylvanica* under field conditions in Poland. *Hymenoscyphus albidus* did not express any perceivable pathogenicity on both host species.

## Introduction

The genus *Fraxinus* comprises 48 tree and shrub species indigenous to the temperate and subtropical regions of the Northern Hemisphere [1]. In Europe, *F*. *excelsior* L. is the most widespread native species, while *F*. *angustifolia* Vahl and *F*. *ornus* L. thrive in Southern Europe. The American green ash (*F*. *pennsylvanica* Marsh.) is the most widely distributed ash species in North America and is also frequently planted in Europe where it has become locally naturalized. Green ash is used for ornamental purposes [2, 3], for timber production [4–6], as part of shelterbelts [7], or as an ameliorative pioneer for fostering the establishment of native trees [2]. In some European countries, green ash is considered an invasive tree species, existing mainly in floodplain forests and along rivers [5, 6, 8].

Since the early 1990s, European ash (*F*. *excelsior*) has been undergoing dieback on a large scale in Europe, calling into question both the survival and future use of this species [9–12]. The causal agent of this epidemic is the ascomycete *Hymenoscyphus fraxineus* (T. Kowalski) Baral, Queloz & Hosoya (= *Hymenoscyphus pseudoalbidus* Queloz et al., anamorph *Chalara fraxinea* T. Kowalski) [13, 14]. The pathogen was introduced from East Asia where it occurs on *Fraxinus mandshurica* Rupr. and *F*. *chinensis* ssp. *rhynchophylla* (Hance) E. Murray (syn. *F*. *rhynchophylla* Hance) [15–17]. The fungus infects ash leaves by means of ascospores and generates necrotic lesions, as well as causing the shedding of leaves. Ascomata are formed in the leaf litter during the subsequent summer. On susceptible hosts, the fungal mycelium can spread from the petiole into the shoot, where it causes extensive xylem and bark necroses eventually leading to tree mortality [14].

*Fraxinus angustifolia* Vahl is also affected by the disease, albeit to a lesser extent, whereas *F*. *ornus* seems to be largely tolerant to the fungus. For the introduced *F*. *pennsylvanica*, field observations of natural infections in several European countries indicated that this species is less susceptible to ash dieback compared to the European species *F*. *excelsior* and *F*. *angustifolia* [14, 18–20]. This was corroborated by stem inoculations in a phytotrone [21]. However, to our knowledge, no results of field inoculations on *F*. *pennsylvanica* have been reported this day.

The ash dieback pathogen, *H*. *fraxineus*, is, morphologically speaking, almost indistinguishable from *Hymenoscyphus albidus* (Roberge ex Gillet) W. Phillips, the existence of which has been known in Europe for over 150 years [22–24]. This species also infects common ash leaves and forms apothecia on the leaf remnants found on the ground through summer; however, it is not regarded as pathogenic [21, 25–27].

Determining whether *F*. *pennsylvanica* could replace *F*. *excelsior* or not is of great significance to European forestry. In North America, neither *H*. *fraxineus* nor *H*. *albidus* are currently present, but both fungi could be introduced in future, thus reinforcing the need for the timely provision of data for the purpose of risk assessment. Therefore, the aim of the present study is to determine the virulence (i.e. the degree of pathogenicity) of both *Hymenoscyphus* species on *F*. *pennsylvanica* under field conditions in Poland and to compare it with the interaction of these fungi with *F*. *excelsior*.

## Materials and Methods

The tests were conducted on an experimental plot in the Stary Sącz Forest District, Southern Poland (49° 33' 83'' N, 20° 39' 35'' E), on five-year-old *Fraxinus excelsior* seedlings (provenance Poland) and also on those of *F*. *pennsylvanica* (provenance unknown). Permission to conduct the field experiments was obtained from the head forester, Mgr inż. Paweł Szczygieł. Inoculations were performed with six and nine *H*. *fraxineus* isolates from Southern Poland [28] on stems and rachises, respectively, as well as three isolates of *H*. *albidus* from Switzerland ([24], see Table 1). The strains are deposited in the culture collection of the University of Agriculture in Cracow.

**Table 1:** Origin of the *H*. *fraxineus* and *H*. *albidus* strains used for the inoculation experiments. All strains were obtained from *F*. *excelsior*.

Isolate No.	Species	Location	Coordinates	Year of inoculation
20011	*H*. *fraxineus*	PL, Siewierz	50° 28' N 19° 14' E	2012
20018	*H*. *fraxineus*	PL, Siewierz	50° 28' N 19° 14' E	2012
20045	*H*. *fraxineus*	PL, Świerklaniec	50° 26' N 18° 56' E	2012
20047	*H*. *fraxineus*	PL, Świerklaniec	50° 26' N 18° 56' E	2012
20058	*H*. *fraxineus*	PL, Brynek	50° 31' N 18° 44' E	2012
20075	*H*. *fraxineus*	PL, Brynek	50° 31' N 18° 44' E	2012
20192	*H*. *fraxineus*	PL, Stary Sącz	49° 33' N 20° 38′ E	2013
20220	*H*. *fraxineus*	PL, Stary Sącz	49° 33' N 20° 38′ E	2013
20227	*H*. *fraxineus*	PL, Ojców	50° 13' N 19° 49' E	2013
090726.5	*H*. *albidus*	CH, Quinto	46° 30' 14.8'' N 08° 42' 59.5'' E	2012, 2013
090812.3	*H*. *albidus*	CH, Leukerbad	46° 22' 55.3'' N 07° 37' 20.1'' E	2012, 2013
090812.7	*H*. *albidus*	CH, Leukerbad	46° 22' 55.3'' N 07° 37' 20.1'' E	2012, 2013

For inoculum production, the fungi were grown for three weeks in darkness at room temperature on malt extract agar (MEA; 20g ⁄ l malt extract, Difco, Sparks, MD, USA; 15g ⁄ l agar, Difco). Subsequently, for *H*. *fraxineus*, small sterile ash wood sticks (5 x 2 x 2 mm) were placed on the colonies and incubated for three additional weeks. For *H*. *albidus*, which is only known to occur on leaves, fragments of ash rachises of similar size were used. For control inoculations, sterile sticks, which had been incubated on MEA for three weeks, were used.

The stems of the current season’s growth (0.6–0.8 cm thick) were inoculated in late July 2012, as were the rachises in 2012 and 2013 (Table 1). The inoculum was inserted into superficial tissue incisions as described in [29] and covered by parafilm^TM^ (Bemis Company, www.parafilm.com). Control inoculations were made in the same manner with sterile inocula. In total, 36 stems and 54 rachises of each *Fraxinus* species were inoculated with both species of *Hymenoscyphus* respectively (Tables 2 and 3).

**Table 2:** Necrotic lesions on rachises two months post inoculation with *H*. *albidus* and *H*. *fraxineus*.

Fungus	Host	Inoculated rachises [n]	With necrotic lesion [n]	Mean length of necrosis* [cm] (min–max)
*H*. *albidus*	*F*. *excelsior*	54	9	0.1 c** (0–1.2)
*H*. *fraxineus*	*F*. *excelsior*	54	54	8.4 a (1.7–16.6)
Control	*F*. *excelsior*	8	0	0.0 c (0)
*H*. *albidus*	*F*. *pennsylvanica*	54	18	0.2 c (0–1.3)
*H*. *fraxineus*	*F*. *pennsylvanica*	54	54	1.9 b (0.5–6.2)
Control	*F*. *pennsylvanica*	8	0	0.0 c (0)

* mean of all rachises

**Variables with the same letter are not significantly different for p = 0.05 (Kruskal-Wallis test).

**Table 3:** Presence of stem necroses 12 months post inoculation with *H*. *albidus* and H. *fraxineus*.

Fungus	Host	Inoculated stems (n)	Lack of longitudinal necrosis [n]		Longi-tudinal necrosis present [n]	Mean length of necrosis* [cm] (min–max)
			complete scarring	incom-plete scarring		
*H*. *albidus*	*F*. *excelsior*	36	23	13	0	0 b** (0)
*H*. *fraxineus*	*F*. *excelsior*	36	0	0	36	18.0 a (8.0–27.9)
Control	*F*. *excelsior*	6	6	0	0	0 b (0)
*H*. *albidus*	*F*. *pennsylvanica*	36	27	9	0	0 b (0)
*H*. *fraxineus*	*F*. *pennsylvanica*	36	25	9	2	1.9 b (1.2–2.7)
Control	*F*. *pennsylvanica*	6	6	0	0	0 b (0)

*–mean of the all isolates

**Variables with the same letter are not significantly different for p = 0.05 (Kruskal-Wallis test).

For *H*. *albidus*, 12 replicate inoculations were made per fungal isolate on rachises in 2012 and six in 2013. Stem inoculations were also performed in 12 replications in 2012.

For *H*. *fraxineus*, six rachises and stems, respectively, were inoculated with the same fungal strain. Each inoculation was carried out on a different tree.

The symptoms on rachises were assessed after two months and those on the stems after 12 months. The lengths of superficially visible necrotic lesions, minus the length of the inoculation wound, were measured and any instances of fungal fructification were recorded. In cases where the entire distal part of the organ had died off in both the rachises and the stems, only the axial extension of the primary necrosis (identifiable by virtue of its distinguished tissue discoloration and depression) was determined.

Reisolations were attempted from all stems and rachises with necrotic lesions (both fungus and mock inoculated) and from plants where wound closure was incomplete (but showing no bark necroses) within 24 hours of harvesting [29]. Six tissue samples were taken from each necrotic stem or rachis—two from the inoculation site, two from the proximal lesion edge and two from an intermediate position in the distal part of the lesion. In the case of partially closed wounds, the samples were taken from below the wound surface and from green tissues approximately 1.5 cm distal and proximal to the inoculation wound. The samples (size approx. 5 x 2 x 2 mm; in the case of very little necroses consisting of dead and living host tissue) were excised aseptically upon disinfection of the surface with 96% ethanol and upon removal of the superficial tissue. The samples were placed on malt extract agar (MEA) with 200mg/L Tetracycline (Tetracyclinum TZF Polfa, Poland) and incubated at 15°C for at least three weeks and identified morphologically. The isolation from an organ was regarded as positive if the respective fungus grew from at least one tissue sample. In total, 1362 inoculation samples and 168 control samples underwent evaluation.

For statistical purposes, one-way analysis of variance (ANOVA) was used, followed by a HSD Tukey test for variables with homogeneity of variance and, in other cases, the non-parametric multiple comparison Kruskal-Wallis test was utilized (see results). Homogeneity of variance was tested by means of a Levene's test. All statistical calculations were performed using the software STATISTICA, version 10 (www.statsoft.com).

## Results

Once *F*. *excelsior* and *F*. *pennsylvanica* were inoculated with *H*. *albidus* and *H*. *fraxineus*, necrotic lesions developed around the inoculation wounds in varying degrees depending on the fungal species tested, the ash species in question and the organ inoculated.

### Rachis inoculations with *H*. *albidus*

**Fig 1:**
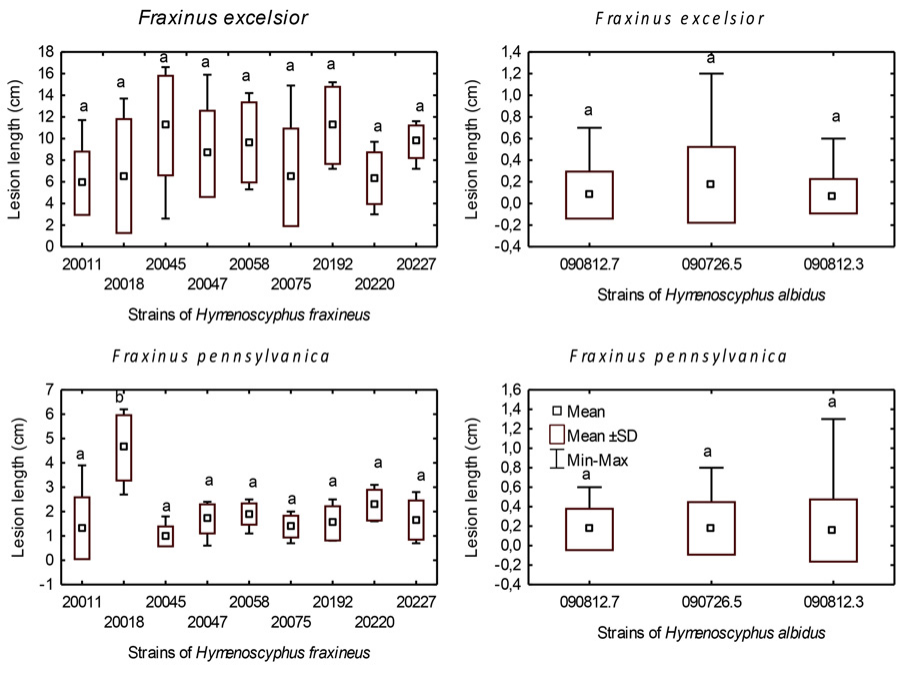
Lesion development on rachises. Difference in necrosis length on *F*. *excelsior* and *F*. *pennsylvanica* rachises two months after inoculation of selected strains of *H*. *fraxineus* and *H*. *albidus*. Variables with the same letter are not significantly different for p = 0.05 (HSD Tukey post hoc test after ANOVA).

**Fig 2:**
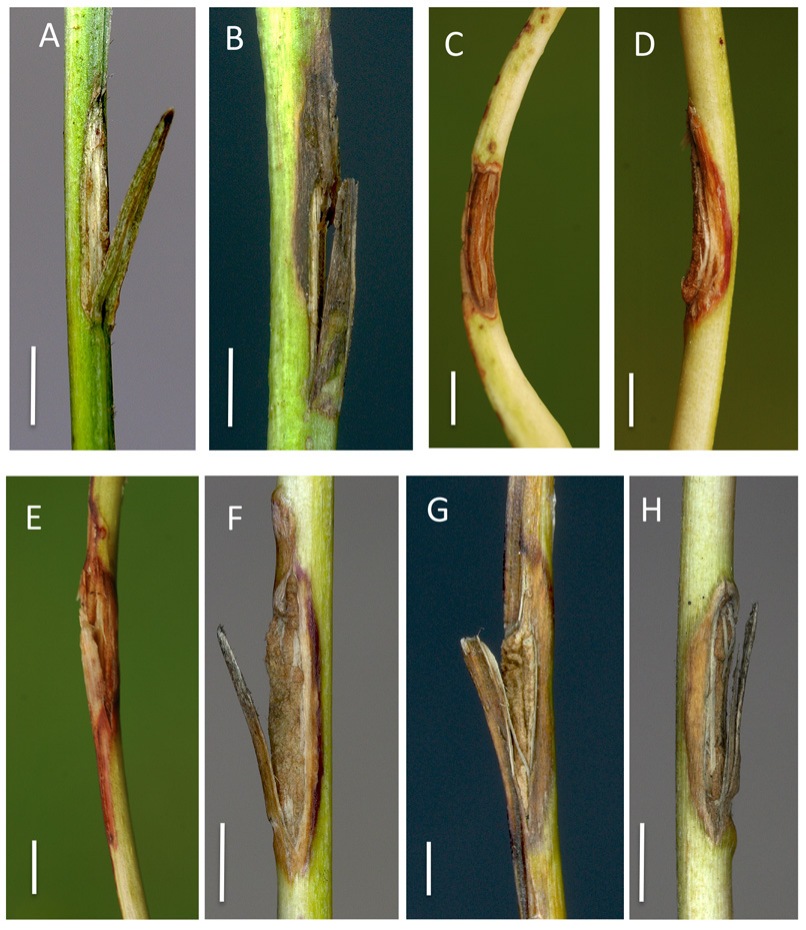
Rachis inoculations with *H*. *albidus* on *F*. *excelsior* (A-B) and *F*. *pennsylvanica* (C-H). Appearance of the inoculated wounds after two months. (A) Wound without callus formation and no necrosis, (B) necrotic lesion starting from the distal wound edge, (C) largely closed wound with no discoloration of the surrounding tissue, (D) completely closed wound surrounded by a narrow area of reddish discoloration, (E) partially closed wound surrounded by a pronounced area of reddish discoloration, (F) completely closed wound surrounded by a narrow necrotic lesion, (G) completely closed wound surrounded by an extended necrotic lesion, (H) partially closed wound with limited necrosis next to the margin. Scale bars denote a length of 0.3 cm.

On rachises inoculated with *H*. *albidus*, we saw necrotic lesions develop on 16.7% of the wounds on *F*. *excelsior* and on 33.3% of those on *F*. *pennsylvanica* (Table 2). However, the necroses were small in size (up to 1.2 cm long on *F*. *excelsior* and 1.3 cm on *F*. *pennsylvanica*) (Table 2, Figs 1 and 2). There was no significant difference in lesion length between the tested isolates (Fig 1). Wound closure was complete in 19.4% of the rachises of both *Fraxinus* species (Fig 2C and 2D). However, in some cases, necroses developed before wound closure could take place (Fig 2F) or, in other cases, were associated with pronounced callus formation (Fig 2G). In the case of *F*. *pennsylvanica*, independent of the presence of a necrotic lesion, reddish discoloration of the rachis surface was observed around the wound in 22.2% of inoculations (Fig 2D–2F).

### Stem inoculations with *H*. *albidus*

**Fig 3:**
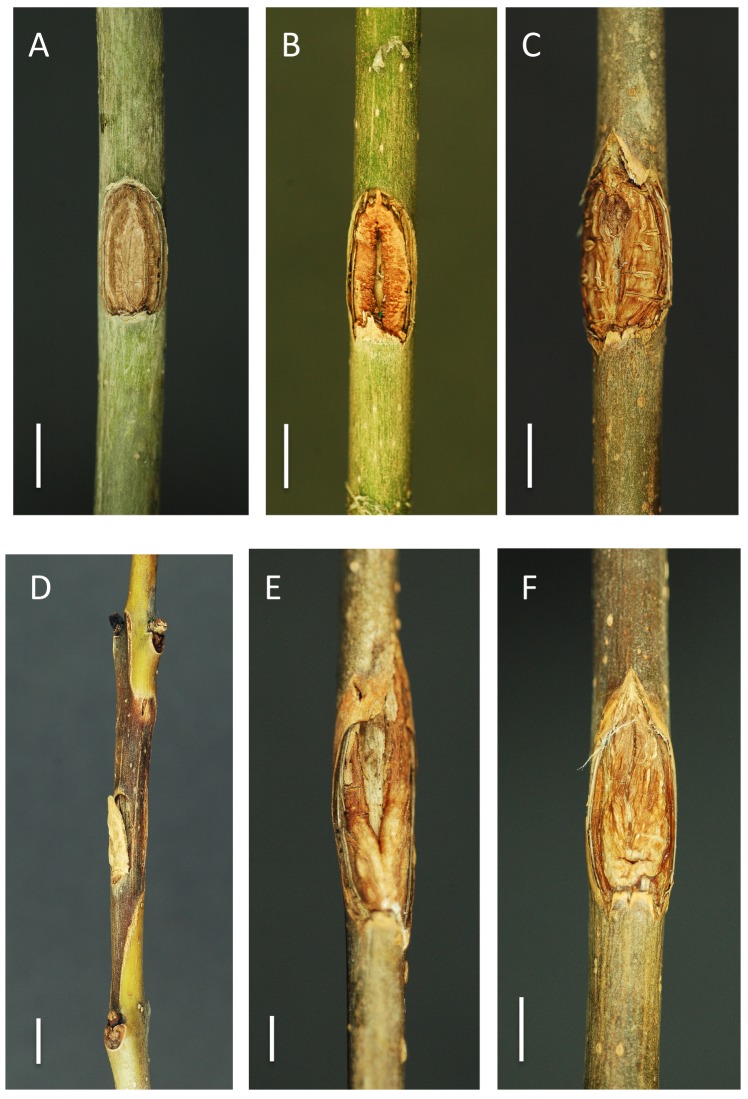
Stem inoculations with *H*. *albidus* (A-C) and *H*. *fraxineus* (D-F). Appearance of the inoculated wounds after 12 months. (A) Completely closed wound on *F*. *excelsior*, (B) partially closed wound on *F*. *excelsior*, (C) completely closed wound on *F*. *pennsylvanica*, (D) extensive necrosis on *F*. *excelsior*, (E) partially closed wound with small necrotic lesion on *F*. *pennsylvanica*, (F) completely closed wound on *F*. *pennsylvanica*. Scale bars denote a length of 0.5 cm.

Stem inoculation with *H*. *albidus* did not produce necrosis on any of the 72 individual trees for either of the two ash species and the inoculation wounds completely or, in some cases partially healed within one year (Fig 3A–3C, Table 3).

### Rachis inoculations with *H*. *fraxineus*

**Fig 4:**
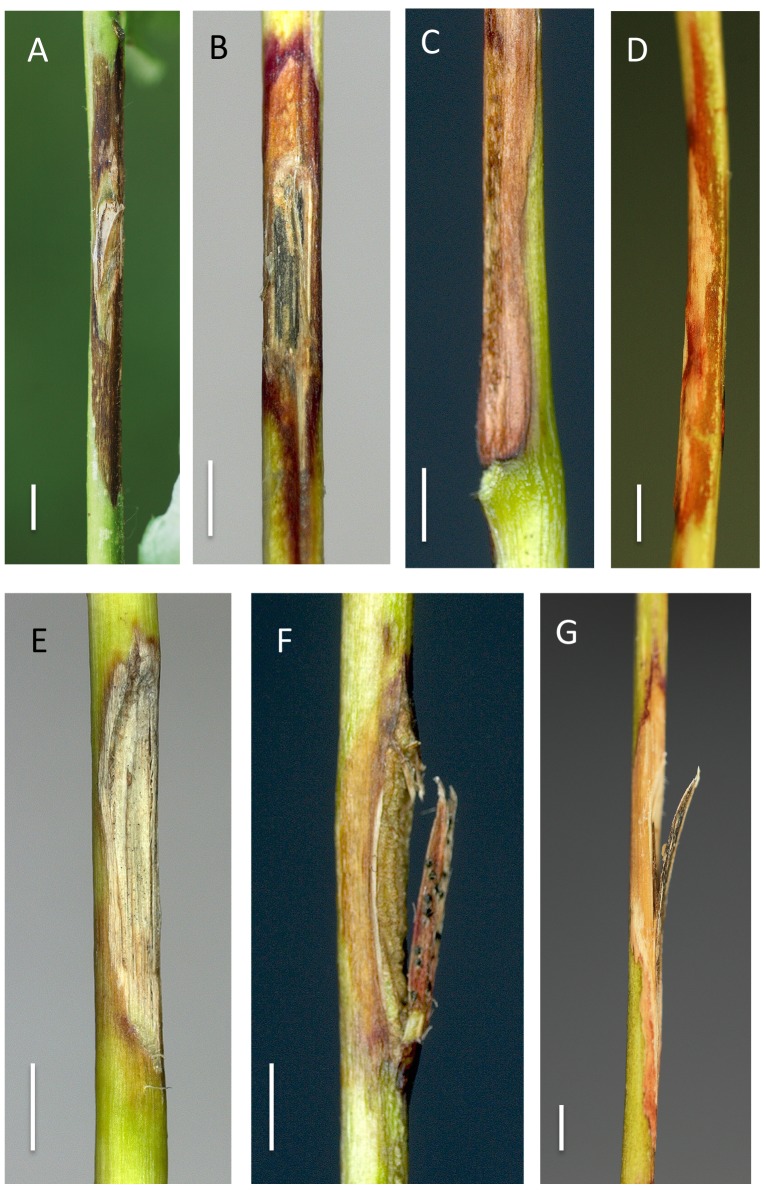
Rachis inoculations with *H*. *fraxineus* on *F*. *excelsior* (A) and *F*. *pennsylvanica* (B-G). Appearance of the inoculated wounds after two months. (A) Extensive necrosis on *F*. *excelsior*, (B) necrosis on *F*. *pennsylvanica* surrounded by reddish discoloration, the wound surface is blackened due to the formation of a pseudosclerotial plate of the fungus, (C) no further growth of the necrosis upon insertion of a petiolule, (D) necrotic lesion surrounded by reddish discoloration, (E) non-necrotic wound without surrounding discoloration, (F) completely closed wound with pycnidia of *Phoma* sp. on the protruding tissue resulting from the incision, (G) extensive necrotic lesion with some reddish discoloration at the distal and proximal ends. Scale bars denote a length of 0.3 cm.

*Hymenoscyphus fraxineus* caused large, light to dark brown necrotic lesions to appear on all inoculated *F*. *excelsior* rachises (mean length 8.4 cm, maximal length 16.6 cm; Table 2, Fig 4A) without exhibiting significant differences between the isolates (Fig 1) or between the samples from different years. The leaf section distal to the lesion had died off at the time of harvest (two months post inoculation) in 35.2% of the rachises. The extension of the necrosis in both proximal and distal directions varied considerably and, in some cases, ceased upon insertion of leaflet petiolules.

Similiarly, on *F*. *pennsylvanica*, necroses also developed on all inoculated rachises (Fig 4B–4G). However, these lesions were significantly smaller than those seen on *F*. *excelsior* (average length 1.9 cm, maximal length 6.2 cm) and considerably larger than those appearing on the control trees (Table 2). Amongst the fungal isolates, strain No. 20018 produced notably longer lesions (HSD Tukey test, p = 0.05) (Fig 1). In a single case, the formation of a pseudosclerotial plate of *H*. *fraxineus* (identified by the presence of *Chalara* phialides) could be observed on the wound surface (Fig 4B). As with *F*. *excelsior*, sometimes the spreading of the lesion was interrupted by leaflet insertions (Fig 4C). The rachis necroses on *F*. *pennsylvanica* were often (64.8% of cases) encased in a reddish discolored area (Fig 4B and 4D). Occasionally single leaflets distal to the lesion withered and, in one case, the whole distal leaf portion died off.

### Stem inoculations with *H*. *fraxineus*

**Fig 5:**
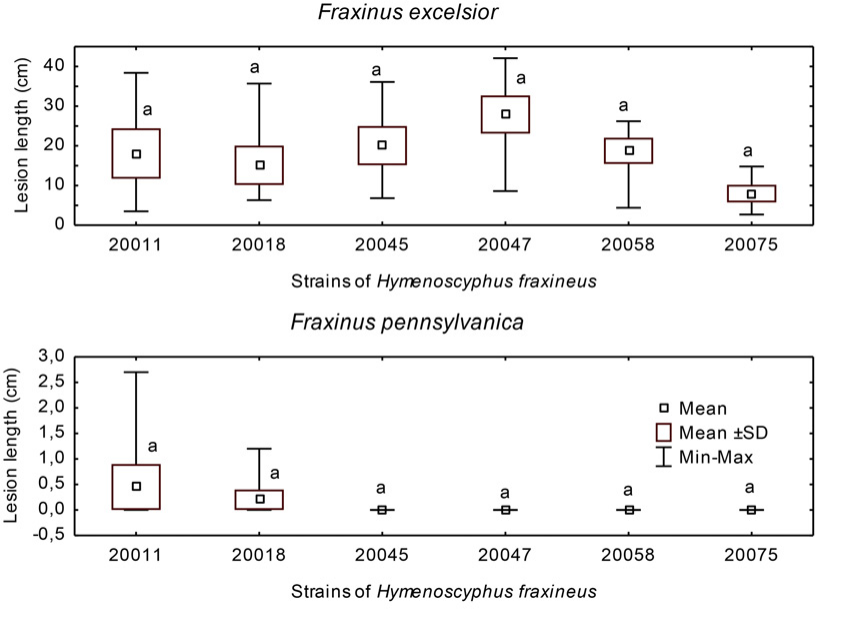
Lesion development on stems. Difference in necrosis length on *Fraxinus excelsior* and *Fraxinus pennsylvanica* stems 12 months after inoculation with selected strains of *Hymenoscyphus fraxineus* and *Hymenoscyphus albidus*. Variables with the same letter are not significantly different for p = 0.05 (Kruskal-Wallis test).

*Hymenoscyphus fraxineus* caused necrotic lesions on all inoculated *F*. *excelsior* stems. Their mean length was 18.0 cm, the maximum being 27.9 cm (Table 3, Figs 5 and 3D). As a result of girdling, the entire distal segments died off in 11.1% of the stems. In contrast, only two out of the 36 (5.5%) inoculated *F*. *pennsylvanica* plants developed necrotic lesions (mean length 1.9 cm, Figs 5 and 3E, Table 3). In all other cases, the wounds either completely or partly healed (Fig 3F) and the distal part of the shoot remained healthy and unharmed. There was no significant difference between the inoculated trees and the control ones (Table 3). The difference in necrosis length between *F*. *excelsior* and *F*. *pennsylvanica* was highly significant. With *F*. *pennsylvanica*, there was no significant difference between the inoculated trees and the control ones (Table 3).

### Reisolations and controls

*H*. *albidus* was only reisolated from some ofvthe inoculated rachises affected by necrosis and from stems showing incomplete wound closure (Table 4) and never from living tissues extending below or above the wound. In contrast, *H*. *fraxineus* was detected in the majority of inoculated rachises of both ash species and in the stems of *F*. *excelsior*, but only very rarely in the stems of *F*. *pennsylvanica* (Table 4). However, fungi of the mitosporic genera *Alternaria*, *Cladosporium*, *Colletotrichum*, *Epicoccum*, *Fusarium*, *Lecytophora*, *Phoma* and *Phomopsis* were repeatedly isolated from necrotic areas or asymptomatic tissues adjacent to the wound. Several species (*Alternaria*, *Cladosporium*, *Phoma*, *Phomopsis*) had sporulated on the dead tissue at the time of sampling (Fig 4F).

**Table 4:** Reisolations of *H*. *albidus* and *H*. *fraxineus* from the wounds of inoculated stems and rachises.

Fungus	Host	Stems		Rachises	
		with necroses % (n) ^1^	with incomplete wound closure % (n)	with necroses % (n)	with in-complete scarring % (n)
*H*. *albidus*	*F*. *excelsior*	-^2^	7.7 (13)	11.1 (9)	9.1 (11)
*H*. *fraxineus*	*F*. *excelsior*	66.7 (36)		70.4 (54)	
Control	*F*. *excelsior*	0 (6)		0 (8)	
*H*. *albidus*	*F*. *pennsylvanica*	-	11.1 (9)	11.1 (18)	8.3 (12)
*H*. *fraxineus*	*F*. *pennsylvanica*	50.0 (2)	11.1 (9)	59.3 (54)	
Control	*F*. *pennsylvanica*	0 (6)		0 (8)	

^1^ Number of samples

^2^ No necroses observed and no reisolations attempted.

**Fig 6:**
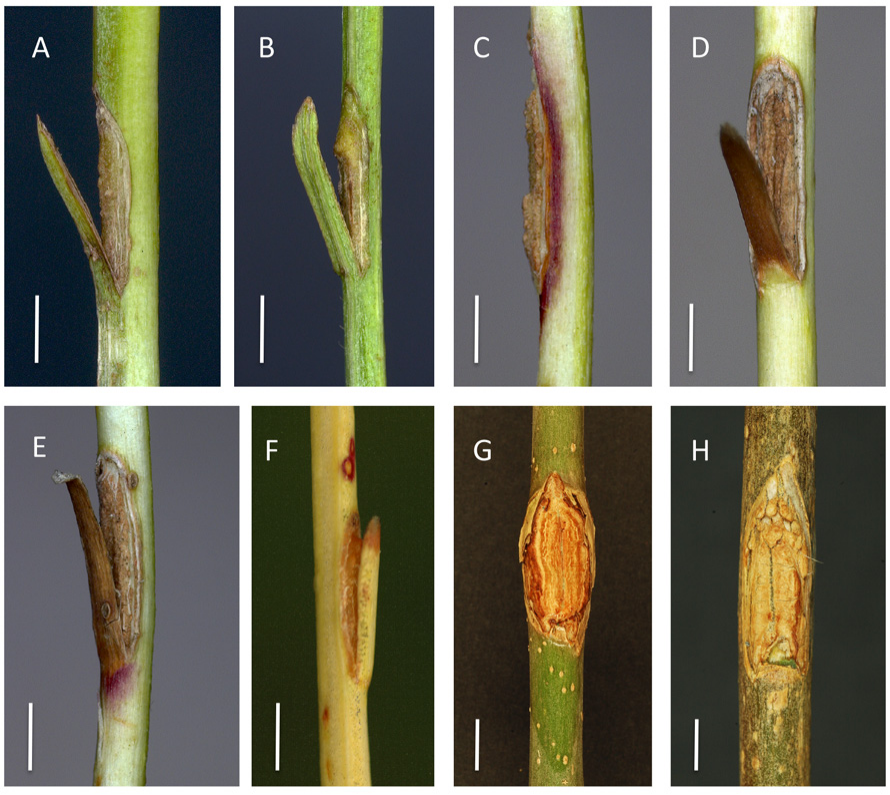
Control inoculations on rachises (A-F) and stems (G-H) of *F*. *excelsior* and *F*. *pennsylvanica*. Appearance of the wounds after two months (rachises) and 12 months (stems). (A-B) completely closed wounds on *F*. *excelsior*, (C) completely closed wound with strong callus formation, surrounded by reddish discoloration on *F*. *pennsylvanica*, (D) normal wound closure with no discoloration, (E) same situation as in D, but with localized reddish discoloration, (F) wound closed by means of an inconspicuous callus, no discoloration, (G) completely closed stem wound on *F*. *excelsior*, (H) completely closed stem wound on *F*. *pennsylvanica*. Scale bars denote a length of 0.3 cm.

None of the *Hymenoscyphus* species were isolated from any of the mock inoculations, independent of host species and organ (Fig 6). All control wounds had completely healed at the time of evaluation (Table 4, Fig 6).

## Discussion

The presented study confirms the high virulence of *H*. *fraxineus* in relation to *F*. *excelsior*. Moreover, we detected no difference in virulence among the fungal strains tested on this host. This uniformity could be explained by the limited allelic diversity of the pathogen population in Europe [15, 21]. The fungus infects several *Fraxinus* species and *F*. *excelsior* is known to be particularly susceptible [14, 30–32]. The encounter of an exotic pathogen with a naive (i.e. non-coevolved) host population can result in the emergence of a new lethal disease. Several such events have previously affected forest tree species populations on a large scale, e.g. chestnut canker, Dutch elm disease, sudden oak death and white pine blister rust [33–37]. A very minor proportion of *F*. *excelsior* exhibits a tolerance to to ash dieback, thus hope remains for the evolutionary rescue of the host in the long run [32]. However, the introduction of additional fungal alleles from Asia could possibly negate this effect [15].

The formation of necrotic lesions caused by *H*. *fraxineus* was repeatedly demonstrated via artificial stem inoculations [19, 29, 38, 39]. We also observed necroses on ash leaf rachises, which led to the dying off of the distal leaf parts, a symptom commonly observed in nature. However, natural infection occurs via ascospores [40] and it is not possible to adequately imitate this process by means of wound inoculations. After ascospore infection, various processes could be considered decisive for disease expression: (i) subtle defense responses of the host during early stages of the infection process and/or (ii) interactions between the pathogen and epiphytic or endophytic leaf colonizers. With our inoculation technique, we focused on capturing the defense capabilities exhibited by the host when confronted with a previously established infection. The fungal metabolites, which are ultimately responsible for necrosis formation, have yet to be defined. Several secondary metabolites of *H*. *fraxineus* have been described [26, 41–46], but their role in necrosis formation is as yet unclear. Currently, the only known *H*. *fraxineus* phytotoxins—viridiol and 3,4-dimethylpentan-4-olide—are also produced by its non-pathogenic sister species *H*. *albidus* [26, 46].

*Hymenoscyphus fraxineus* induced significantly smaller lesions on *F*. *pennsylvanica* compared to those that formed on *F*. *excelsior* and our data are well in line with the observations that green ash is significantly less susceptible to ash dieback than *F*. *excelsior*. Also, on this host, there were almost no differences in virulence among the fungal strains, only one of the nine strains caused significantly larger necroses (Fig 1). Concerning the susceptibility of *F*. *pennsylvanica* to *H*. *fraxineus*, only a small number of coincidental observations are available: In Estonia, *F*. *pennsylvanica* is affected to a moderate degree [18]. In Austria, dieback of *F*. *pennsylvanica* has only been observed sporadically and this species appears to be much more resistant than both *F*. *excelsior* and *F*. *angustifolia* [20]. In northern Germany, only mild symptoms were noted subsequent to exposure to natural infection pressure [19]. However, following stem wound inoculations with *H*. *fraxineus* in a phytotron, *F*. *pennsylvanica* exhibited a moderate level of susceptibility. In addition, a relatively high proportion (16% out of 75) of natural stem infections was observed on plants delivered from a nursery in Germany [21]. As a consequence, we cannot discount the importance of environment-related predisposition with regards to this host’s susceptibility [47] or the possibility that particularly susceptible provenances exist [48]. The native range of green ash is vast and the species comprises at least three ecotypes [49]. A significant drawback of our data, as well as of the studies cited above, is the fact that no detailed information on host provenance is available. Future susceptibility tests should therefore be performed on a more representative selection of *F*. *pennsylvanica* genotypes.

For *F*. *pennsylvanica*, in contrast to *F*. *excelsior*, the susceptibility of rachises was much higher than that of stems, but the extension of the necroses along rachises took shape more slowly than they did on *F*. *excelsior*, indicating the presence of an effective defense mechanism. Consequently, the risk of stem infection is considerably smaller for *F*. *pennsylvanica* than for *F*. *excelsior*. This gives rise to the possible solution of replacing, to an extent, infested *F*. *excelsior* with *F*. *pennsylvanica* in Europe. However, this only applies should the expansion of the emerald ash borer in Europe be contained [50, 51].

Neither *H*. *fraxineus* nor *H*. *albidus* have been detected in North America [52]. The introduction of *H*. *fraxineus* to North America would add to the threat already being posed by the emerald ash borer. Since green ash, like other ash species, could serve as a vector for the ash-dieback pathogen, plant transfer from Europe or Asia to North America should be avoided. Other American ash species are also at risk, such as *F*. *nigra* Marsh., which has proven highly susceptible to *H*. *fraxineus* in Estonia [18]. Our experiments demonstrate that *H*. *albidus*, which is indigenous to Europe, is virtually non-pathogenic for both ash species, independent of the inoculated organ (rachis or stem). Only small necrotic lesions developed on a handful of artificially inoculated ash rachises and *H*. *albidus* was seldom reisolated. Consequently, we cannot disregard that *H*. *albidus* exerts some influence, albeit minor, on the tissues surrounding the inoculation wound, potentially predisposing them to secondary fungi. albudus

This fungus is evidently particukarly poorly adapted to the wound environment or, alternatively, it does not compete well against other wound colonizing microbes. *Hymenoscyphus albidus* was never isolated from living tissue and this could cast doubts on the possibility of this species leading an endophytic lifestyle, as suggested by Baral und Bemmann [23]. Nevertheless, molecular detection techniques could possibly reveal the presence of the fungus in living plant tissue, as shown by Cleary et al. [53] for *H*. *fraxineus* in ash seeds. So far, *F*. *excelsior* rachises (and in rare cases those of *F*. *angustifolia*) have been recognized as a substrate of *H*. *albidus* [23, 54–56]. The sporulation window of *H*. *albidus* spans July to September [23, 56]. Our inoculations were made using colonized rachis pieces at the beginning of this period, most likely providing a suitable precondition for fungal development. In any case, our data show that the ecological behaviour of *H*. *albidus* does not mirror that of *H*. *fraxineus* after wound inoculation and further detailed studies of its biology are required. In comparison with *H*. *fraxineus*, such data might enhance our understanding of ash dieback etiology. In spite of the limitations discussed above, we conclude that *H*. *albidus* does not pose a noteworthy risk for *F*. *pennsylvanica* and the same has also recently been concluded for *F*. *mandshurica* [27]. However, the *Hymenoscyphus* species comprise only a minute proportion of the whole tree microbiome and ecological surprises could definitely arise [57]. Avoidance of further pathogen introductions and a comprehensive understanding of host-pathogen interactions in a changing environment represent the greatest modern-day challenges for forest pathology [58].
